# Vitamin C may reduce troponin and CKMB levels after PCI and CABG: a meta-analysis

**DOI:** 10.1186/s12872-023-03459-6

**Published:** 2023-09-21

**Authors:** Sander Rozemeijer, Harri Hemilä, Marlinde van Baaren, Angélique M.E. de Man

**Affiliations:** 1 Department of Intensive Care Medicine, Research VUmc Intensive Care (REVIVE), Amsterdam Cardiovascular Science (ACS), Amsterdam Infection and Immunity Institute (AI&II), Amsterdam Medical Data Science (AMDS), Amsterdam UMC, Location VUmc, Vrije Universiteit Amsterdam, De Boelelaan 1117, Amsterdam, 1081 HV The Netherlands; 2 Department of Anesthesiology, Amsterdam UMC, Location VUmc, Vrije Universiteit Amsterdam, De Boelelaan 1117, Amsterdam, 1081 HV The Netherlands; 3 Department of Public Health, University of Helsinki, Helsinki, Finland

**Keywords:** Vitamin C, Ascorbic acid, Ischemia/reperfusion injury, Periprocedural myocardial injury, PMI, Cardiac enzymes, Troponin, CKMB, PCI, CABG

## Abstract

**Background:**

Ischemia/reperfusion injury contributes to periprocedural myocardial injury (PMI) in patients undergoing percutaneous coronary intervention (PCI) or coronary artery bypass grafting (CABG). PMI can be estimated by the elevation of troponin (Tn) and creatine kinase-MB (CKMB) plasma levels, and it is associated with increased risk of cardiovascular events and mortality. Vitamin C might have a beneficial effect on PMI by improving endothelial function, improving myocardial perfusion, and by reducing oxidative stress generated during/after reperfusion. In several small animal models of cardiac stress, vitamin C reduced the increase in Tn and CKMB levels. The aim of this meta-analysis was to investigate whether vitamin C administration may have an effect on Tn and CKMB levels in patients undergoing PCI or CABG.

**Methods:**

We searched PubMed, Cochrane, Embase and Scopus databases for controlled clinical trials reporting on Tn and CKMB levels in adult patients who underwent PCI or CABG and received vitamin C. As secondary outcomes we collected data on biomarkers of oxidative stress in the included trials. In our meta-analysis, we used the relative scale and estimated the effect as the ratio of means.

**Results:**

We found seven controlled trials which included 872 patients. All included trials administered vitamin C intravenously, with a range from 1 to 16 g/day, and all initiated vitamin administration prior to the procedure. Vitamin C decreased peak Tn plasma levels in four trials on average by 43% (95% CI: 13 to 63%, p = 0.01) and peak CKMB plasma levels in five trials by 14% (95% CI: 8 to 21%, p < 0.001). Vitamin C also significantly decreased the biomarkers of oxidative stress.

**Conclusions:**

Vitamin C may decrease cardiac enzyme levels in patients undergoing elective PCI or CABG. This may be explained partially by its antioxidant effects. Our findings encourage further research on vitamin C administration during cardiac procedures and in other clinical contexts that increase the level of cardiac enzymes. Future studies should search for an optimal dosing regimen, taking baseline and follow-up plasma vitamin C levels into account.

**Supplementary Information:**

The online version contains supplementary material available at 10.1186/s12872-023-03459-6.

## Introduction

In patients with acute myocardial infarction (MI) or angina pectoris treated with percutaneous coronary intervention (PCI) or coronary artery bypass grafting (CABG), ischemia/reperfusion injury contributes to periprocedural myocardial injury (PMI). It has been estimated that PMI occurs in up to 30–40% of the patients treated with PCI [1–3] and CABG [4], and is associated with increased risk of cardiovascular events and mortality [2–7]. The extent of PMI can be estimated by the elevation of the levels of cardiac enzymes such as creatine kinase-MB (CKMB), though troponin (Tn) is nowadays preferable as it is more sensitive and specific [2–4].

In several small animal models of cardiac toxicity, vitamin C administration attenuated the rise of troponin and CKMB [8–10]. In addition, vitamin C reduced infarct size in animal studies when administered alone [11], in its oxidized form, as dehydroascorbic acid [12], and when combined with vitamin E [13]. These pre-clinical findings suggest that in cardiac stress vitamin C might also have protective effects on the myocardium in humans. Vitamin C plays an important role in preserving endothelial function [14]. As an indication of clinical relevance of such biochemical effects, vitamin C improved endothelial function in atherosclerotic and heart failure patients [15]. Moreover, vitamin C improved the perfusion of the myocardium in patients undergoing PCI and people exposed to hyperoxia [16–18]. Recent meta-analyses found that vitamin C improved left ventricular ejection fraction (LVEF) in cardiac and non-cardiac patients [19], and prevented post-operative atrial fibrillation (POAF) in high risk patients in trials carried out outside of the USA [20].

One of the factors that contributes to ischemia/reperfusion injury is the increase in reactive oxygen species (ROS) [21], mostly arising directly after reperfusion [22]. Oxidative stress may cause reversible or irreversible injury to proteins, lipids and DNA [23]. Decreased plasma vitamin C concentrations are often reported after MI and cardiac surgery, accompanying the increase in oxidative stress [24–26], which indicates that the consumption of vitamin C is increased. As a result, the decreased vitamin C levels might impair its pleiotropic effects [27] and may further be harmful as the protection against the remaining oxidative stress can become inadequate. The potential antioxidant effects on PMI should be investigated further [28].

The aim of this meta-analysis was to investigate whether vitamin C administration may have an effect on Tn and CKMB levels in patients undergoing PCI or CABG.

## Methods

### Selection criteria for studies

We included controlled clinical trials investigating the effect of vitamin C on Tn and CKMB levels in adult patients who underwent PCI or CABG. We did not restrict to randomized trials. We restricted to trials in which vitamin C was the only difference between the trial groups. Administration of placebo to the control group was not required. We included both oral and intravenous vitamin C administration. We did not set limits on the duration of vitamin C administration.

### Search strategy

PubMed, Cochrane, Embase and Scopus were searched for eligible studies published up to August 9th 2023. The search consisted of the terms vitamin C, troponin and CKMB (see Additional File 1 for the search terms). Moreover, the references of included studies and relevant reviews were checked for additional studies.

### Outcomes

The primary outcomes of our analysis are peak plasma levels of Tn and CKMB. As secondary outcomes we collected data about biomarkers of oxidative stress.

### Selection of studies and data extraction

We excluded duplicates through Rayyan software. Two independent investigators (SR and MvB) screened each study for eligibility against the inclusion criteria. When title or abstract suggested a paper might meet inclusion criteria, the full text of these potentially eligible studies was retrieved and reviewed. Any disagreement concerning the eligibility of particular studies was discussed with a third reviewer (AdM).

### Quality assessment of the trials

The quality of the included studies was evaluated by two reviewers (SR and HH) using the criteria outlined in the Cochrane Handbook for Systemic Reviews of Interventions [29]. The following criteria were assessed: random sequence generation, allocation concealment, blinding of participants and personnel, blinding of outcome assessment, incomplete outcome data and selective reporting. Disagreements were resolved by consensus. Each quality entry was judged as low, unclear or high risk of bias.

### Statistical methods

In our statistical analysis, we used the relative scale and calculated the ratio of means (RoM) as the measure of effect [30]. There was substantial variation in the timing of Tn and CKMB measurements between the studies, see Table 1. If several time points were available, we chose the peak levels in both groups, regardless of the time point at which the peak level was measured. If vitamin C has an effect on the cardiac enzyme levels, it is possible that the timing of peak is changed together with the height of the peak levels. Therefore, selecting the same time point from curves may not be the best comparison.

**Table 1:** Characteristics of included studies

Trial [ref]	Country	Treatment setting	Age (yr), mean	*N* (vit C/ Control)	Vitamin C administration			Outcome	Timing measurement
					Dose (g/d)	Duration (days)	Timing		
Wang [31]	China	Elective PCI	58	265/267	3	1	Prior PCI	TnI CKMB	Baseline, 6 and 24 h after PCI^a^
Antonic [44]	Slovenia	CABG	65	52/53	2–4	6	Around surgery^b^	TnI	ICU-admission and 18 h after surgery
Dingchao [34]	China	CPB (diagnosis unknown)	-	45/40	16	1	Around surgery^c^	CKMB	13 time points up to 48 h after surgery
Basili [16]	Italy	Elective PCI	67	28/28	1	1	Prior PCI	TnI	Baseline and every 6 h after PCI over the next 2 days^a^
Emadi [60]	Iran	CABG	62	25/25	10	1	Around surgery^d^	TnI CKMB	During induction of anesthesia, end of bypass, 6 h and 24 h after surgery
Oktar [33]	Turkey	CABG	56	12/12	4	1	Prior surgery	CKMB	13 time points up to 72 h after surgery
Demirag [45]	Turkey	CABG	64	10/10	6.5	1	Around surgery^e^	CKMB	Baseline and 2 h after surgery

**Abbreviations: CKMB: Creatine kinase-MB; CPB: Cardiopulmonary bypass; PCI: Percutaneous coronary intervention; Tn: Troponin**

^a^ Not all time points were reported individually

^b^ 2 times 2 g vitamin C prior to surgery and 1 gram twice a day for 5 days after the surgery

^c^ 125 mg/kg 30 min before surgery and 125 mg/kg at aortic declamping (each time). Assumed weight: 65 kg

^d^ 5 g of vitamin C prior to the surgery and 5 g in the cardioplegic solution

^e^ 50 mg/kg after the induction of anaesthesia and 50 mg/kg just before aortic declamping. Assumed weight: 65 kg

Additional file 2 (spreadsheet) shows our calculations. Two trials that were included in the meta-analysis did not report the mean and standard deviation (SD) values [16, 31]. We estimated the mean from the reported median and interquartile range [IQR] [32], see Additional file 1 and 2. We used the reported p-values to calculate the standard error (SE) for the log(RoM) for these two trials [16, 31]. In the Oktar trial [33] there were 2 different groups of patients receiving vitamin C. In group II, vitamin C was administered intravenously prior to the induction of anesthesia and in group III, vitamin C was added to the cardioplegic solution. In our analysis, we used the patients receiving intravenous vitamin C (group II) as the intervention group, because vitamin C was initiated earlier and there was more time for the vitamin to distribute in the tissues before the operation. As a sensitivity analysis, we also carried out the meta-analysis with the vitamin C group III, but the result did not differ considerably, see Additional file 1. The CKMB plasma levels of the Dingchao trial [34] were measured from a published figure by using a graphics program; see Additional File 2. In our analyses, we concluded that the dispersion parameter of the CKMB levels in the Oktar and Dingchao trial was SE, and not SD as was reported, see Additional file 1 and 2.

We pooled the included trials with the *metagen* function of the R package *meta* [35, 36]. We used the inverse variance fixed effect options. Our calculations are shown in Additional file 1.

## Results

### Description of the included trials

**Fig. 1:**
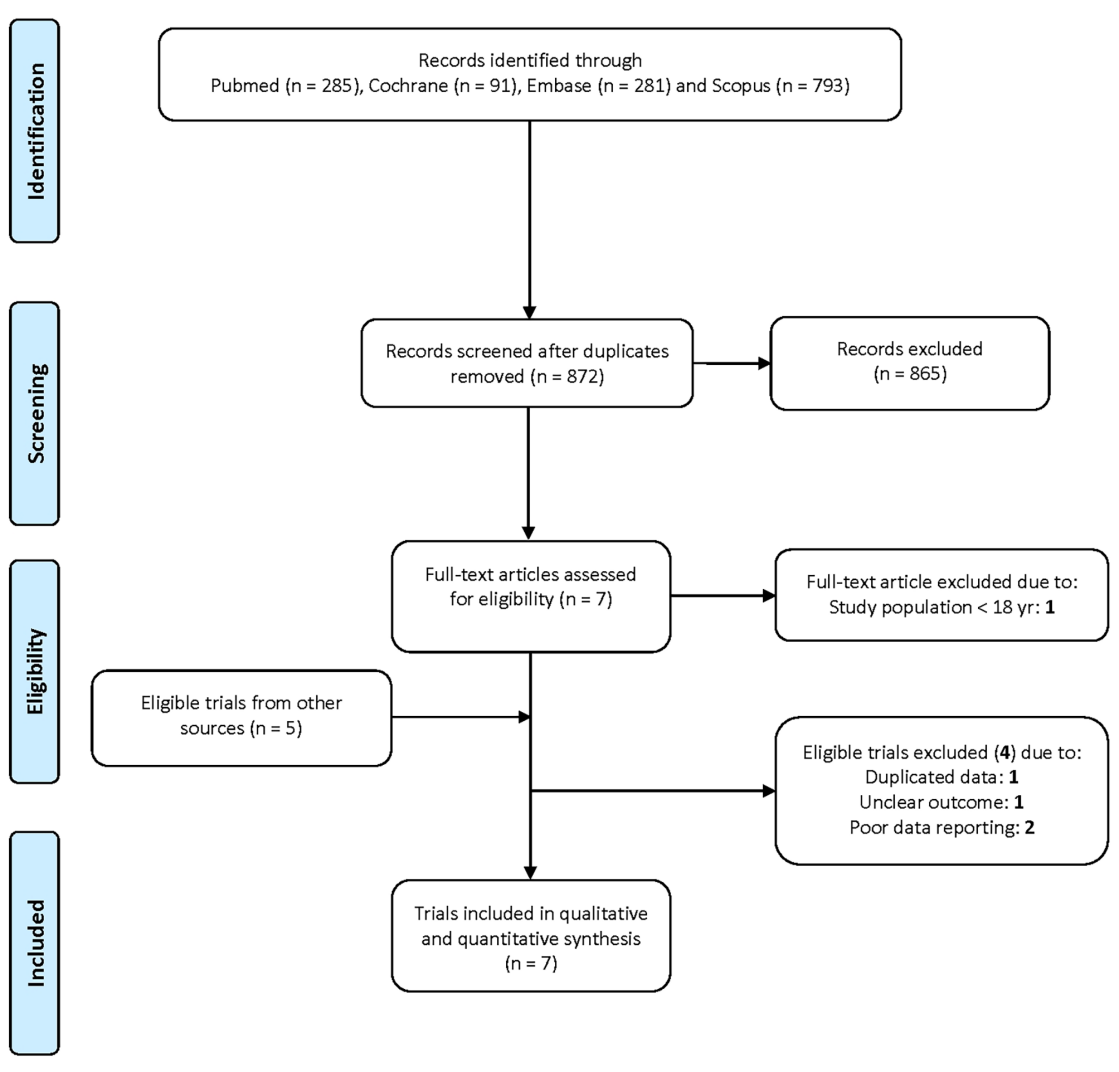
PRISMA flow diagram of the searches. Search terms are described in Additional file 1

We found 11 potentially eligible trials on the effect of vitamin C on plasma levels of Tn and/or CKMB in patients at risk for PMI (Fig. 1). Four trials were excluded due to suspected data duplication [37, 38], unclear troponin-related outcome [39] and poor data reporting in general [40–43]. Seven trials were included in the meta-analysis of which four trials reported the changes of TnI and five of CKMB (Table 1). A detailed description of all included trials and a summary of the problems of the excluded trials is available in Additional file 1.

In total 872 patients were included in the seven trials. In six trials patients underwent elective PCI (N = 588) or CABG (N = 199). In one trial, the type of cardiac surgery was unknown of 85 patients who underwent cardiopulmonary bypass (CPB) [34]. The mean age of the patients ranged from 56 to 67 years. The doses of vitamin C ranged from 1 to 16 g/day. All seven included trials administered vitamin C intravenously (i.v.) and vitamin C administration was initiated prior to the procedure. The duration of vitamin C administration was one day in six trials, and six days in one trial [44]. The first day was the day of the procedure. The timing of measuring plasma levels of troponin and CKMB was substantially different among the trials (Table 1). Two trials measured cardiac enzymes at more time points than were reported [16, 31].

**Fig. 2:**
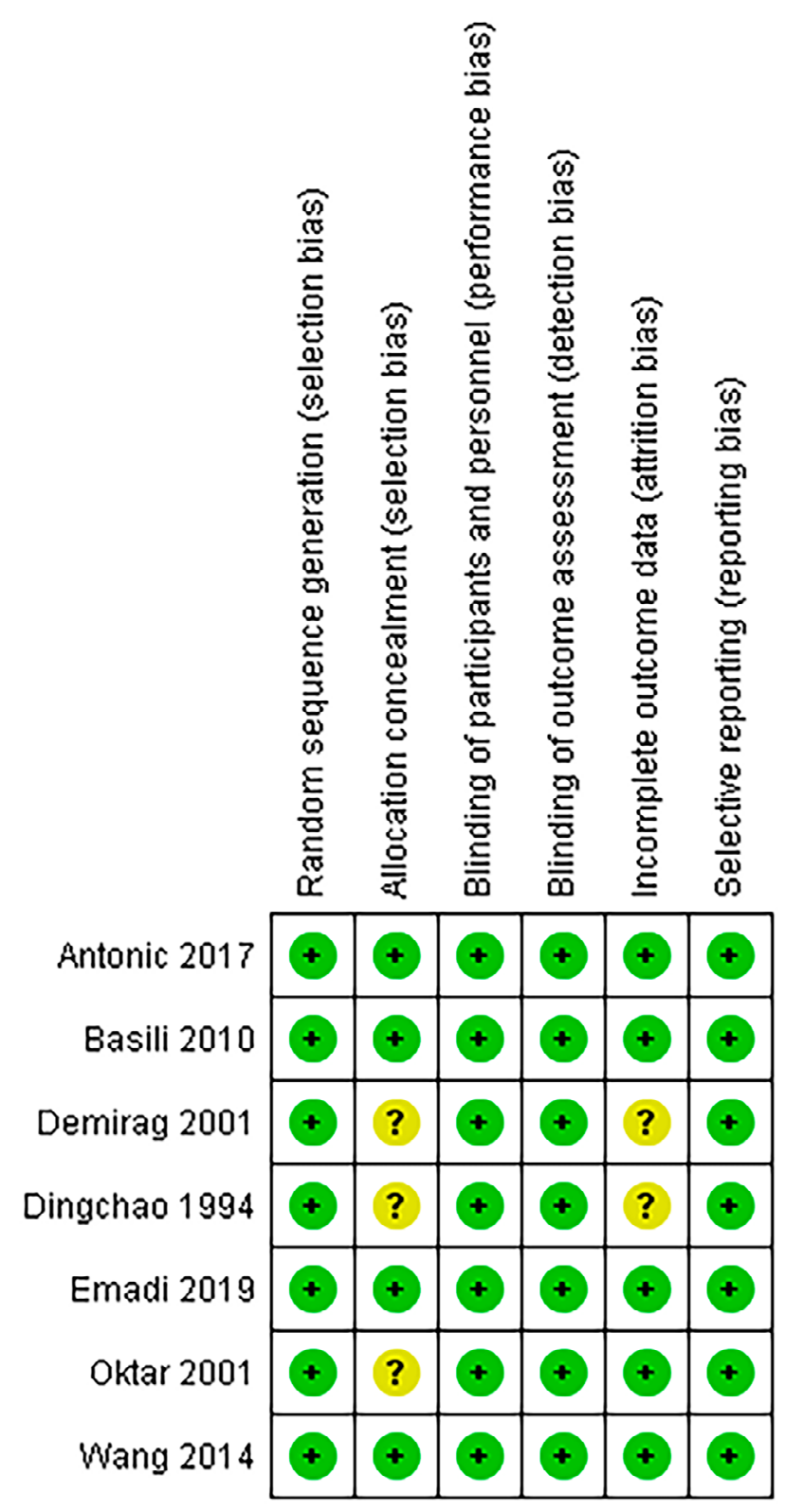
Risk of bias summary. Review authors’ judgements about each risk of bias item for each included study. A green plus sign (+) indicates ‘low risk’ for bias. A question mark (?) indicates ‘some concerns’ for bias or that conclusions are unable to be drawn regarding potential bias. The reference numbers to the trials are shown in Table 1. In Additional File 1 the support for the assessments can be found

The risk of bias assessment of the included studies is shown in Fig. 2. Five trials were randomized. One randomized trial [45] and two other trials [33, 34] did not describe the method of allocation. However, Oktar [33] and Dingchao [34] both showed that the groups were balanced for baseline characteristics, and for baseline CKMB and malondialdehyde (MDA) levels. In these three trials there was no information about the blinding of allocation stage, treatment and outcome assessment [33, 34, 45]. However, since our primary outcome is a laboratory measure, we judged that there is low risk of performance and detection bias caused by possible lack of blinding [46].

### Effect of vitamin C on cardiac enzyme levels

In our statistical analysis, we used the relative scale and calculated the ratio of means (RoM) [30] as the measure of effect. For example, in the vitamin C group of the Oktar trial [33], the peak CKMB level in the vitamin C group was 64 U/L after CABG, and in the control group 89.33 U/L. As a result, the RoM is 0.72 (64/89.33), which corresponds to a 28% lower peak level of CKMB in the vitamin C group.

**Fig. 3:**
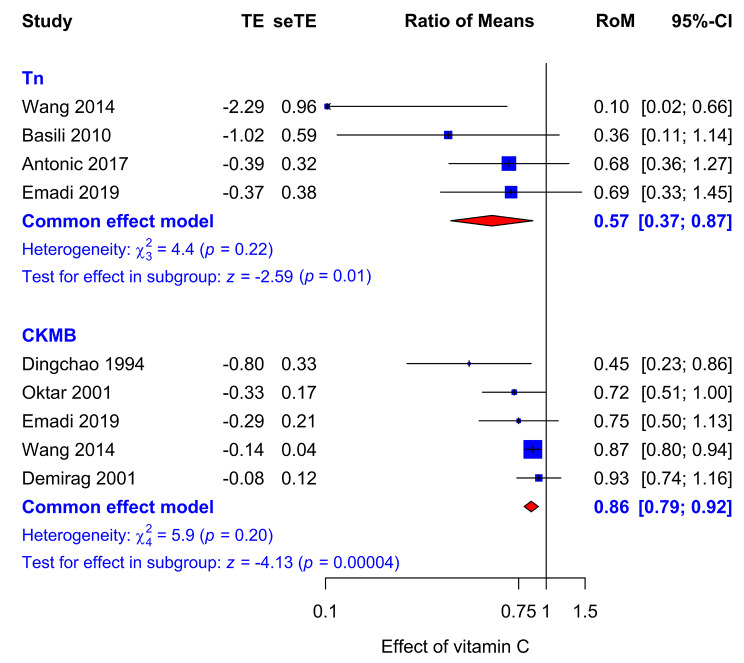
Effect of vitamin C on cardiac enzyme levels. The upper model shows the effect of vitamin C on Tn and the lower model shows the effect of vitamin C on CKMB. The effect of vitamin C is presented as the RoM with 95% CI. The horizontal lines indicate the 95% CI ranges and the blue squares indicate the point estimate of the effect in that particular trial. Treatment effect (TE) indicates ln(RoM). The size of the blue square reflects the weight of the trial in the meta-analysis. The diamond indicates the pooled effect and 95% CI for the Tn and CKMB subgroup. See Additional File 1 for the description of the trials and calculations

In a group of four trials, vitamin C significantly decreased peak TnI plasma levels on average by 43% (95% CI: 13 to 63%, p = 0.01), see Fig. 3. One of the troponin trials found a significant effect within the trial. There was no substantial heterogeneity between the trials (p = 0.2). In a group of five trials, vitamin C significantly decreased CKMB plasma levels on average by 14% (95% CI: 8 to 21%, p < 0.001). Three of the CKMB studies found a significant effect within the trial. There was no substantial heterogeneity between the trials also for this outcome (p = 0.2). The Wang trial has by far the greatest weight in the CKMB analysis since it has 532 participants. In contrast, the total number of participants in the four other CKMB trials in Fig. 3 is only 179. Therefore, we carried out a sensitivity analysis in which we excluded the Wang trial. The pooled effect of vitamin C on CKMB level remained significant: p = 0.0085 (Additional File 1). The diagnosis in the Dingchao trial was not described and we carried out a sensitivity analysis in which we excluded that trial. The pooled effect of vitamin C on CKMB level remained significant: p = 0.0001 (Additional File 1).

### Comparison of timing and vitamin C plasma levels in the Oktar trial

Oktar reported two groups of patients to whom vitamin C was administered using different protocols. Group II received intravenous vitamin C just before the induction of anesthesia, and group III received the vitamin in the cardioplegic solution. The achieved peak plasma vitamin C concentrations were quite similar but the timing of the vitamin C peak was later in group III [33].

**Fig. 4:**
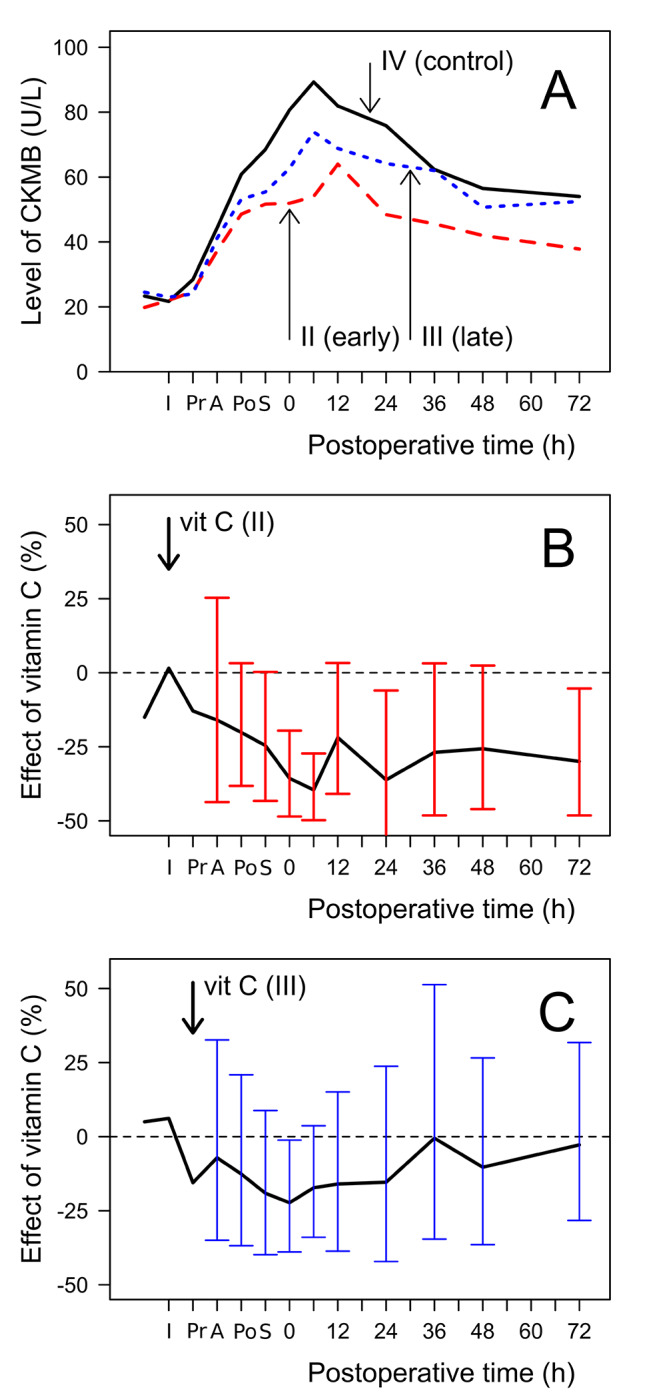
Effect of vitamin C on CKMB levels in two vitamin C groups in Oktar et al. [33]. **A.** Course of CKMB plasma level in early-vitamin C group II (red dashed line), late-vitamin C group III (blue dotted line) and control (black continuous line). Effect of vitamin C on CKMB plasma levels in group II (**B.**) and group III (**C**.) versus control. Abbreviations: I: Induction; Pr: PreCPB; A: After declamping; Po: PostCPB; S: Skin closure

We analyzed the time pattern of the CKMB levels in the Oktar et al. trial (Fig. 4). Compared with the control group, the CKMB levels in the two vitamin C groups are consistently lower over the follow-up period (Fig. 4A). In the vitamin C group II, who received vitamin C earlier, the peak CKMB plasma level was lower and at a later timepoint compared with vitamin C group III. We also compared both vitamin C groups against the control group. In the early-vitamin C group II, there were four time points after the operation at which the CKMB levels were significantly lower compared with the control group (Fig. 4B). In the late vitamin C group III, there was only one time point after the operation at which the CKMB levels were significantly lower than the control group (Fig. 4C). Although this indicates that there is stronger evidence of benefit from the earlier vitamin C administration, the confidence intervals of the two vitamin C groups are extensively overlapping and thus the difference between the two administration methods has not been demonstrated. Nevertheless, the difference gives more motivation for further research on the effects of timing.

### Effect of vitamin C on biomarkers of oxidative stress

Basili et al. [16] reported that 1 h after balloon inflation the serum level of 8-OHdg, an oxidative stress marker, was decreased in the vitamin C group by 38% (95% CI 26–48%) (Table 2). Wang et al. [31] reported that at 6–8 h after PCI, the 8-OHdG serum level was decreased in the vitamin C group by 41% (95% CI 38–45%) (Table 2). Basili also reported a 63% (95% CI 39–78%) decrease in the level of another oxidative stress marker, 8-iso-PGF_2α_.

**Table 2:** Effect of vitamin C on biomarkers of oxidative stress after procedure

Outcome	Trial (ref.)			
		Vit C	Control	RoM (95% CI)
8-OHdG (ng/mL)	Basili [16]	2.6 ± 1.1^a^	4.2 ± 1.1	0.62 (0.52–0.74)
	Wang [31]	2.4 ± 1.0^b^	4.1 ± 1.1	0.59 (0.55–0.62)
8-iso-PGF_2α_ (pg/mL)	Basili [16]	50 [20.7-102.5]^c^	161.5 [117.5–190.0]	0.37 (0.22–0.61)^d^

**Abbreviations: 8-iso-PGF**_**2α**_ **: 8-iso-prostaglandin F2alpha; 8-OHdG: 8-hydroxy-2’-deoxyguanosine**

The figures are described as means ± SD or median [IQR] when appropriate

^a^ p < 0.001, ^b^ p < 0.001 and ^c^ p < 0.0001 for the differences between vitamin C and control group (as reported in the papers)

^d^ Means were estimated from the median and the quartiles [32]. The reported p-value was used to calculate the SE of ln(RoM). See calculations in Additional file 2

A third marker for oxidative stress that has been measured in the included trials is MDA. Dingchao et al. [34], Oktar [33] and Demirag [45] found significantly lower levels of MDA after the operation in the vitamin C group compared to the respective control group. Dingchao [34] and Oktar [33] measured the MDA levels at multiple time points after the procedure and found significantly lower MDA levels in the vitamin C group until 9 h and 1 day postoperative, respectively.

## Discussion

In this meta-analysis we found that on average vitamin C reduced post-operative Tn plasma levels by 43% and CKMB plasma levels by 14% in patients who underwent elective PCI or CABG. The significant reduction in cardiac enzyme levels indicate a parallel reduction in PMI.

There are multiple ways in which vitamin C may exert cardioprotective effects [27]. Vitamin C can directly scavenge harmful oxidative stress, restore other antioxidants and antioxidant enzymes, and decrease ROS production [47]. Vitamin C significantly lowered biomarkers of oxidative stress in all included trials that reported them [16, 31, 33, 34, 45], but further research is needed on oxidative stress biomarkers and PMI [28]. Vitamin C is consumed when there is overwhelming oxidative stress. Furthermore, when recycling of vitamin C is insufficient, its level may decrease systemically or locally, thereby impairing the pleotropic effects of vitamin C [27]. Vitamin C may further exert a cardioprotective effect by preserving endothelial function as has been found in multiple clinical contexts [14, 15, 48–50]. There is also evidence suggesting that vitamin C can improve myocardial perfusion [16–18].

Some reports in the older literature suggested that vitamin C deficiency, scurvy, may be associated with chest pain and abnormal ECG changes, which were reversed after vitamin C administration [51–53]. These early observations also indicate that vitamin C has effects on heart functions. In a further early study, vitamin C was effective in reducing creatine kinase levels in patients undergoing CABG with > 50 min ischemic time, whereas the vitamin had no effect on patients with < 50 min ischemic time [54].

In type II diabetic patients with cardiovascular risk and in surgical patients with limb ischemia/reperfusion injury vitamin C administration significantly reduced Tn levels compared to control [55, 56]. A recent trial, however, found no benefit of vitamin C and N-acetylcysteine versus placebo on post-operative myocardial injury in patients undergoing major non-cardiac surgery [57]. Importantly, median Tn level was low in the placebo group. Thus, the lack of effect of vitamin C in this non-cardiac trial might be explained simply by the very low level of cardiac stress. In our study, we investigated the effects of vitamin C in the setting of PCI and CABG, two conditions that uniformly cause cardiac stress and substantial increase cardiac enzyme levels.

In the large PHS-II trial, long-term daily supplementation of 0.5 g/day vitamin C did not reduce the risk of major cardiovascular events [58]. However, the population consisted of well-nourished physicians without prevalent cardiovascular disease. Therefore, the context was very different from the trials included in our analysis. The findings of the PHS-II trial are not a relevant comparison for findings with PCI and CABG patients.

Previous studies have shown that PMI is associated with the incidence of adverse outcomes and the risk of death after PCI and CABG [2–7, 59]. Therefore, reducing prognostically important PMI, especially in high risk patients, might improve patient outcome, e.g. by reducing Type 4–5 myocardial infarction or death [2–4]. Three of the included trials also reported beneficial effects of vitamin C on cardiac function [16, 34, 60]. In addition, a recent meta-analysis found a beneficial effect of vitamin C on LVEF in cardiac and non-cardiac patients [19]. A meta-regression analysis in that study found a highly significant relationship between the baseline LVEF level and the size of effect by vitamin C [19]. Vitamin C had no effect for people with high baseline LVEF level, while the effect was progressively larger with lower LVEF levels. Another meta-analysis in cardiac surgery patients found that vitamin C shortened the length of hospital stay, ICU stay and the occurrence of POAF [20]. However, there was significant heterogeneity in the findings as the benefits were only found in non-United States trials [20]. In our current analysis, all included trials were carried out outside of the USA, and therefore we could not carry out a similar comparison.

The effect of vitamin C may depend on dose, timing, route of administration and the total duration of treatment. In the included trials vitamin C dose ranged from 1 to 16 g at the day of surgery, but no dose-effect was evident. All trials administered vitamin C prior to, and some during, the procedure. One trial had a prolonged treatment for 6 days, however TnI was measured at 18 h [44]. The data from the trial by Oktar et al. suggests that early administration of vitamin C, prior to the procedure, is more beneficial compared to during the procedure (Fig. 4), though this requires further study. Also the optimal duration of vitamin C therapy remains to be investigated as it appears that oxidative stress is increased for multiple days post-operatively [26]. None of the trials administered vitamin C orally, so we could not compare oral and intravenous administration. Absorption of oral doses become saturated at about 3 g/day, whereas i.v. administration can lead to over 100 times higher plasma concentrations [61, 62]. Nevertheless, in the context of PCI and CABG, intravenous administration is convenient as all patients have i.v. routes available because of the procedures. Finally, measuring plasma vitamin C concentrations would add valuable information about size of effect by baseline and follow-up vitamin C status [63, 64].

In addition to differences in dosing regimens, treatment/clinical settings also differed among the included trials. In two trials patients were scheduled for PCI, whereas in five trials patients underwent cardiac surgery. Cardiac surgery may generate higher levels of ROS due to extracorporeal circulation compared to PCI. The size of the effect by vitamin C may differ between the two clinical contexts, but the included trials were too small to compare the two contexts. Oxidative stress parameters such as the static oxidation-reduction potential (sORP) can be measured quickly to quantify the amount of oxidative stress that is generated in different settings [64, 65], and may be used in future research to explore the relation between oxidative stress and PMI [28].

No side effects of vitamin C administration were reported in the included trials. Previous evidence also indicates that both oral and intravenous vitamin C administrations are remarkably safe [66, 67]. The US nutritional recommendations monograph considers various proposed harms (e.g. kidney stone formation, increased oxygen demand and pro-oxidant effects) of high doses of vitamin C, but concluded that the great majority of them are unsubstantiated [66]. Nevertheless, high dose vitamin C may be harmful for the kidneys, but only when administered for a longer period of time or at an extremely high dose. The US recommendations stated that 2 g/day is safe for ordinary people, yet encourages research of higher doses in the contexts of controlled trials [66].

The recently published LOVIT-trial investigated the effect of 4-day vitamin C administration in septic patients [68]. Persistent organ dysfunction and mortality were increased in the vitamin C group. However, the harm occurred after vitamin C was stopped, and not during the vitamin C administration. Therefore, the harm is explained by the rebound effect, and does not indicate harm of ongoing vitamin C administration [69].

### Limitations

The diverse dosing regimens and differences in treatment/clinical settings among the included trials impact the generalizability of the overall conclusions. In addition, the number of included trials is small. Therefore, the evidence we provide does not allow practical conclusions at clinical level. Our goal was to figure out whether the limited number of trials encourage further research and our positive conclusion is not limited by the heterogeneity in dosing regimens and clinical contexts. The blinding of treatment and outcome measures was not described in three included trials [33, 34, 45], but we do not consider that such objective laboratory measures as Tn and CKMB could be meaningfully influenced by the knowledge about the intervention [46]. Therefore, we judged that performance and detection bias were low also for these three trials. Finally, concerns about blinding do not influence our conclusion that this topic should be investigated further. The number of studies and participants was small; however, small size can lead to a false negative finding, but cannot lead to a false positive finding.

## Conclusion

Our study indicates that vitamin C may decrease cardiac enzyme levels in patients undergoing elective PCI or cardiac surgery. Biomarkers of oxidative stress were decreased by vitamin C, suggesting that the effect of vitamin C in the cardiac tissue may be explained, at least in part, through its effect as an antioxidant. The effects of vitamin C administration should be further investigated in cardiac procedures and other clinical conditions in which the levels of cardiac enzymes are increased. Future studies should also search for an optimal dosing regimen, taking baseline and follow-up plasma vitamin C levels into account.

### Electronic supplementary material

Below is the link to the electronic supplementary material.

Additionale File 1: Searches, calculations and detailed descriptions of the included and excluded trials

Additional File 2: Spreadsheat with calculations

## Data Availability

All extracted data and calculations are published in the Additional files.

## List of Abbreviations

8-iso-PGF_2α_: 8-iso-prostaglandin F(2alpha)
8-OHdG: 8-hydroxy-2’-deoxyguanosine
CABG: coronary artery bypass graft
CKMB: creatine-kinase MB
CPB: cardiopulmonary bypass
i.v.: intravenously
IQR: interquartile range
LVEF: left ventricular ejection fraction
MDA: malondialdehyde
MI: myocardial infarction
PCI: percutaneous coronary intervention
PMI: periprocedural myocardial injury
POAF: post-operative atrial fibrillation
RoM: ratio of means
ROS: reactive oxygen species
SD: standard deviation
SE: standard error
sORP: static oxidation-reduction potential
Tn: troponin
