# Supplementary material for: Vitamin C may reduce troponin and CKMB levels after PCI and CABG: a meta-analysis
Source: BMC Cardiovasc Disord. 2023 Sep 21;23:475. doi: 10.1186/s12872-023-03459-6 (PMC10512653; doi:10.1186/s12872-023-03459-6)
Supplement: Supplementary file 1 — Additionale File 1: Searches, calculations and detailed descriptions of the included and excluded trials [file 12872_2023_3459_MOESM1_ESM.pdf]

Vitamin C may reduce troponin and CKMB levels after PCI and CABG: a meta-analysis

Supplementary Material

Sander Rozemeijer, Harri Hemilä, Marlinde van Baaren and Angelique de Man

Source: *BMC Cardiovascular Disorders*. https://doi.org/10.1186/s12872-023-03459-6

Sander Rozemeijer, MD

Department of Intensive Care Medicine, Amsterdam UMC, location VUmc

Vrije Universiteit Amsterdam, De Boelelaan 1117, 1081 HV Amsterdam, The Netherlands

Department of Anesthesiology, Amsterdam UMC, location VUmc

Vrije Universiteit Amsterdam, De Boelelaan 1117, 1081 HV Amsterdam, The Netherlands

E-mail: s.rozemeijer@amsterdamumc.nl

Contents **Page**

Search terms for the database searches	2
Calculations with data of the included trials (explanatory to Excel file)	3
Printouts of statistical calculations, including sensitivity analyses	5
Detailed description of the included and problems of the excluded trials	10

Search terms for the database searches

Pubmed – 285 search results, 2023-08-09

(ascorb* OR "vitamin c") AND (troponin OR "creatine kinase")

Cochrane – 91 search results, 2023-08-09

(("vitamin c" OR ascorb*) AND (troponin OR "creatine kinase" OR CKMB))

Embase – 281 search results, 2023-08-09

('vitamin c':ti,ab,kw OR 'ascorb*':ti,ab,kw) AND ('creatine kinase':ti,ab,kw OR 'troponin':ti,ab,kw OR 'ckmb':ti,ab,kw)

Scopus – 793 search results, 2023-08-09

TITLE-ABS-KEY ((("vitamin c" OR ascorb*) AND (troponin OR "creatine kinase" OR ckmb)))

Other sources

5 reports

Calculations with data of the included trials

In Additional file 2, all calculations can be found of the included trials. In the Notes section of each trials above, the motivation for the data used is decribed. Here, we give explanatory notes on the different worksheets and formulas used in Excel.

Worksheet 1 – Primary endpoint

Worksheet 1 summarizes all data used for our primary endpoint. To calculate RoM, we need means ± SD for each group in each trial. For two trials, Wang and Basili, only skewed data was reported. Therefore, we estimated the mean for each group by adding the first quartile, the mean and the third quartile, and then dividing by 3 (S1). Now the RoM was calculated: the ratio of the means.

For the meta-analysis, the natural logaritm of RoM (treatment effect (TE)) and its standard error (SE) were calculated, according to the literature (S2). To check the correctness of the formula, an example of Friedrich was taken (shown in blue line). When SD was not available, due to skewed data in Wang and Basili, the SE of the TE was estimated by using the reported p-value. In the tabel, the Z-score of these p-values is shown. With the Z-score the SE can be estimated (TE/Z-score = SE). This is shown in the table below. Now all the SE's are estimated, the 95% confidence interval of the RoM can be calculated, as shown in the last two collumns.

Oktar group III was also added, as these data was used in the sensitivity analysis.

Worksheet 2 – Dingchao curve measurement

Dingchao only reported data in a curve. As described above, the nummerical data could be retrieved from the curve with the use of the GIMP graphics program. As a reference, the pixels of 90 and 10 IU/L were estimated first, after which the IU/L of the peaks CKMB could be estimated based on the measured pixels.

This was also done for the CKMB levels at 90 and 120 minutes, as these data will be used on worksheet 3.

Worksheet 3 – Oktar_Dingchao SD

We suspected that the dispersion parameter in the curves of Oktar and Dingchao is SE, not SD as mentioned in the paper.

In Oktar, we recalculated the p-value of the difference in CKMB at 48h (group II-IV) while assuming SD is SE (scenario 1) and SD is SD (scenario 2). The p-value of scenario 2 was very small (1,04243E-09), and the p-value of scenario 1 (p=0.08) was close to the reported p-value of 0.03. This was also concluded for the p-value of the difference at 36h (group II-IV) when assuming SD is SE. Therefore, the reported dispersion of the CKMB results were considered to be SE's. Thus, SE was used to calculate the SD for all time points as shown in Table 2 in this worksheet. The yellow marked data was then used in worksheet 1.

In Dingchao, we recalculated the p-value of the difference in CKMB levels at 90 minutes (non-significant in the paper) while assuming that the reported SD is SE (scenario 1), and while assuming that SD is SD (scenario 2). The data from worksheet 2 was used. The p-value of scenario 2 was very small, and the p-value of scenario 1 was close to what is expected from the curve. This was also concluded for the p-value of the difference at 120 min when assuming SD is SE. Therefore, the peak

CKMB levels and SE was measured from the curve. SE was then used to calculate the SD, which was used in worksheet 1.

Worksheet 4 – Secondary endpoint

For 8-Ohdg and 8-iso-PGF2alpha the RoM with 95% confidence interval was calculated in exactly the same way as described for worksheet 1.

Printouts of statistical calculations

Calculation of the meta-analysis of vitamin C effect on Tn and CKMB

```
> tn <- read.csv("tn.csv")
> tn
        studlab subgroup      TE   seTE
1  Antonic 2017       Tn -0.3890 0.3190
2    Emadi 2019       Tn -0.3665 0.3750
3     Wang 2014       Tn -2.2858 0.9570
4   Basili 2010       Tn -1.0230 0.5907
5 Dingchao 1994     CKMB -0.8041 0.3304
6    Emadi 2019     CKMB -0.2874 0.2106
7    Oktar 2001     CKMB -0.3335 0.1693
8     Wang 2014     CKMB -0.1395 0.0422
9  Demirag 2001     CKMB -0.0771 0.1170
>
>
> tnMA <- metagen(TE, seTE, studlab, data=tn, sm="RR", random=F, subgroup=
subgroup)
> tnMA
Number of studies combined: k = 9

                        RR           95%-CI     z  p-value
Common effect model 0.8454 [0.7859; 0.9093] -4.51 < 0.0001

Quantifying heterogeneity:
 tau^2 = 0.0121 [0.0000; 1.1195]; tau = 0.1099 [0.0000; 1.0580]
 I^2 = 42.0% [0.0%; 73.3%]; H = 1.31 [1.00; 1.93]

Test of heterogeneity:
     Q d.f. p-value
 13.80    8  0.0872

Results for subgroups (common effect model):
                  k     RR           95%-CI    Q   I^2   tau^2     tau
subgroup = Tn     4 0.5670 [0.3693; 0.8705] 4.42 32.1% <0.0001 <0.0001
subgroup = CKMB   5 0.8555 [0.7944; 0.9212] 5.94 32.7% <0.0001  0.0016

Test for subgroup differences (common effect model):
                   Q d.f. p-value
Between groups  3.43    1  0.0639
Within groups  10.36    7  0.1689

Details on meta-analytical method:
- Inverse variance method
- Restricted maximum-likelihood estimator for tau^2
- Q-profile method for confidence interval of tau^2 and tau
>
```

Effect of vitamin C on CKMB: sensitivity analysis to exclude Wang (2014)

```
> ckmb_NoWang <- subset(tn, tn$subgroup == "CKMB" & tn$studlab!= "Wang 2014")
> ckmb_NoWang
        studlab subgroup      TE   seTE
5 Dingchao 1994     CKMB -0.8041 0.3304
6    Emadi 2019     CKMB -0.2874 0.2106
7    Oktar 2001     CKMB -0.3335 0.1693
9  Demirag 2001     CKMB -0.0771 0.1170
>
> ckmb_NoWangMA <- metagen(TE, seTE, studlab, data=ckmb_NoWang, sm="RR", random=F)
> ckmb_NoWangMA
Number of studies combined: k = 4

                         RR           95%-CI     z p-value
Common effect model 0.8003 [0.6780; 0.9446] -2.63  0.0085

Quantifying heterogeneity:
 tau^2 = 0.0199 [0.0000; 1.2319]; tau = 0.1412 [0.0000; 1.1099]
 I^2 = 41.9% [0.0%; 80.5%]; H = 1.31 [1.00; 2.26]

Test of heterogeneity:
    Q d.f. p-value
 5.17    3  0.1599

Details on meta-analytical method:
- Inverse variance method
- Restricted maximum-likelihood estimator for tau^2
- Q-profile method for confidence interval of tau^2 and tau
```

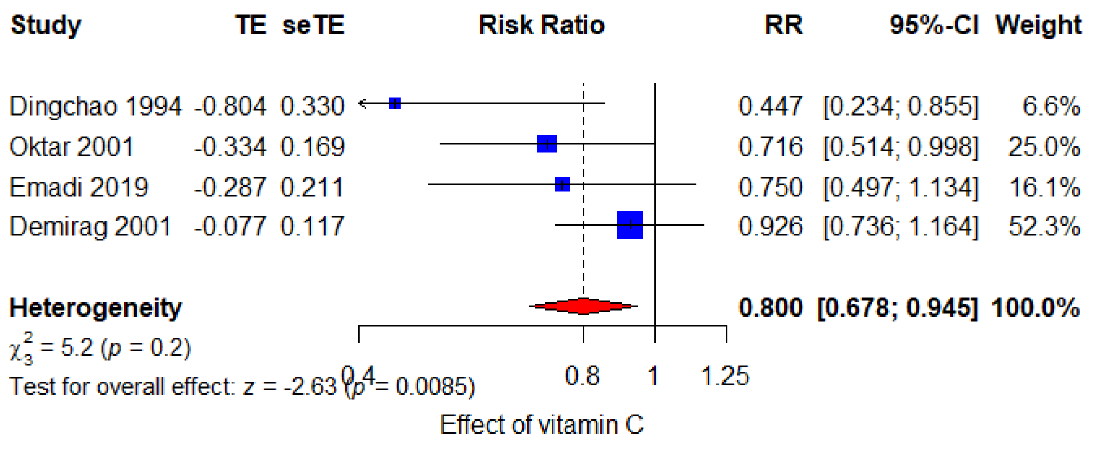

Effect of vitamin C on CKMB: sensitivity analysis to exclude Dinchao (1994)

```
> ckmb_NoDing <- subset(tn, tn$subgroup == "CKMB" & tn$studlab!= "Dingchao 1994")
> ckmb_NoDing
       studlab subgroup      TE   seTE
6   Emadi 2019     CKMB -0.2874 0.2106
7   Oktar 2001     CKMB -0.3335 0.1693
8    Wang 2014     CKMB -0.1395 0.0422
9 Demirag 2001     CKMB -0.0771 0.1170
>
> ckmb_NoDingMA <- metagen(TE, seTE, studlab, data=ckmb_NoDing, sm="RR", random=F)
> ckmb_NoDingMA
Number of studies combined: k = 4

                        RR           95%-CI     z p-value
Common effect model 0.8629 [0.8009; 0.9296] -3.88  0.0001

Quantifying heterogeneity:
 tau^2 = 0 [0.0000; 0.1815]; tau = 0 [0.0000; 0.4260]
 I^2 = 0.0% [0.0%; 84.7%]; H = 1.00 [1.00; 2.56]

Test of heterogeneity:
    Q d.f. p-value
 2.05    3  0.5629

Details on meta-analytical method:
- Inverse variance method
- Restricted maximum-likelihood estimator for tau^2
- Q-profile method for confidence interval of tau^2 and tau
```

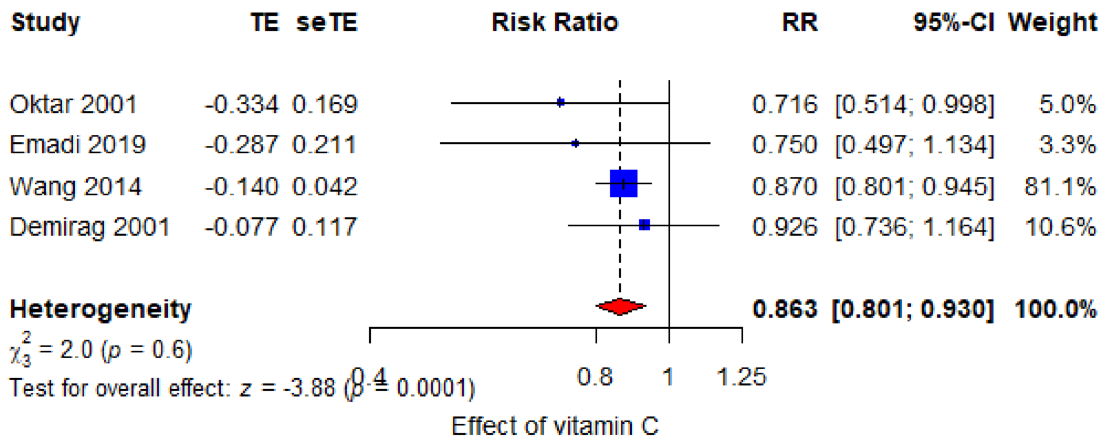

Effect of vitamin C on CKMB: sensitivity analysis using vitamin C group III of Oktar (2001)

```
> ckmb3 <- read.csv("ckmb_oktar3.csv")
> ckmb3
             studlab subgroup       TE    seTE
1       Dingchao 1994     CKMB -0.80413 0.33039
2          Emadi 2019     CKMB -0.28743 0.21055
3 Oktar 2001 (gr. III)     CKMB -0.18940 0.14870
4           Wang 2014     CKMB -0.13947 0.04220
5        Demirag 2001     CKMB -0.07712 0.11695
>
>
> ckmb3MA <- metagen(TE, seTE, studlab, data=ckmb3, sm="RR", random=F)
> ckmb3MA
Number of studies combined: k = 5

                          RR           95%-CI     z  p-value
Common effect model 0.8612 [0.8001; 0.9268] -3.99 < 0.0001

Quantifying heterogeneity:
 tau^2 < 0.0001 [0.0000; 0.6224]; tau = 0.0021 [0.0000; 0.7889]
 I^2 = 17.8% [0.0%; 82.9%]; H = 1.10 [1.00; 2.42]

Test of heterogeneity:
    Q d.f. p-value
 4.87    4  0.3013

Details on meta-analytical method:
- Inverse variance method
- Restricted maximum-likelihood estimator for tau^2
- Q-profile method for confidence interval of tau^2 and tau
```

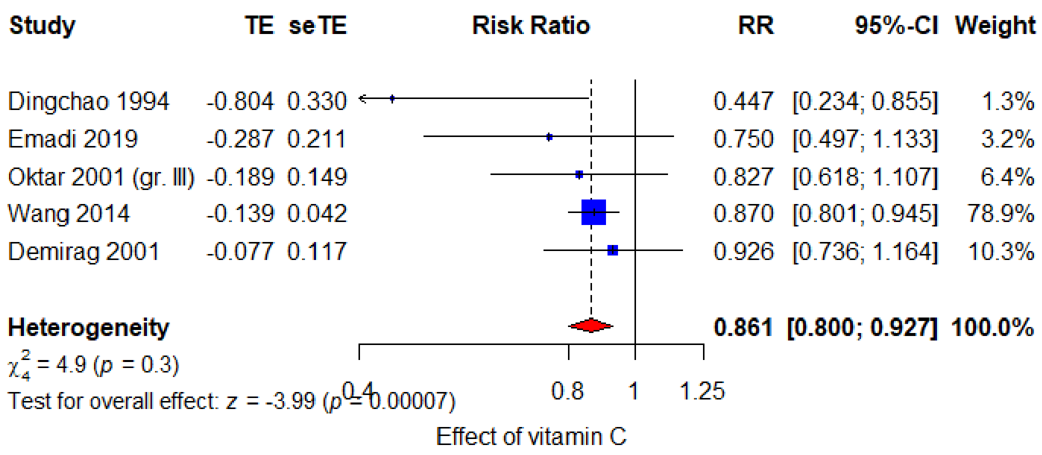

References

S1. Wan X, Wang W, Liu J, Tong T. Estimating the sample mean and standard deviation from the sample size, median, range and/or interquartile range. BMC Medical Research Methodology. 2014;14(1):135.

S2. Friedrich JO, Adhikari NKJ, Beyene J. Ratio of means for analyzing continuous outcomes in meta-analysis performed as well as mean difference methods. Journal of Clinical Epidemiology. 2011;64(5):556-64.

Characteristics of studies

Characteristics of included studies

Antonic 2017

**Methods**	Randomized trial, March 2013 to June 2014<br>https://pubmed.ncbi.nlm.nih.gov/26917198/<br>**DOI:** https://doi.org/10.1016/j.jjcc.2016.01.010<br>Sample size calculation was considered in DOI.
**Participants**	Slovenia, CABG patients, 82 M / 23 F, age: vit C 65 (SD 7) y / control 65 (SD 9) y, 52 vit C / 53 control<br>**Inclusion:**<br>Elective CABG patients<br>**Exclusion:**<br>Emergency operations, any concomitant valve or other surgery, history of AF, pacemaker, history of nephrolithiasis, off-pump surgery.
**Interventions**	**Vit C:**<br>Intravenously 2 g of ascorbic acid 24 h and 2 h prior to surgery.<br>Intravenously 1 g twice a day for 5 days after the surgery.<br>**Control**:<br>Standard care; no mention of saline as placebo.
**Outcomes**	TnI
**Timing of cardiac enzyme measurement**	On ICU-admission and 18 h after surgery
**Notes**	TnI was reported in the result text and Table 3.<br><br>**Tn I:**<br>VitC (18h): 3.26 SD 3.26; Control (18h): 4.81 SD 10.05<br><br>It is puzzling that the SD and mean for vitC groups are identical, but this was confirmed by Dr. Antonic.<br><br>We contacted Dr. Antonic to ask for details about their methods. Contact dates: 2016-11-29 and 2022-01-27.

Risk of bias table

Bias	Authors' judgement	Support for judgement
Random sequence generation (selection bias)	Low risk	"The enrolled patients were then randomly assigned to either the ascorbic acid or control group" (p99).<br>"We used random.org online service" and patients were included in a strict order with no exception. Dr. Antonic provided this information (mail 2016-12-01).<br>The groups were well matched for age, sex, EuroSCORE II (European System for Cardiac Opeative Risk Evaluation), DM2, serum creatinine, preoperative beta-blocker and statin,

		and the number of bypass grafts. (Tables 1 and 2).
Allocation concealment (selection bias)	Low risk	See above.
Blinding of participants and personnel (performance bias)	Low risk	Not described. It seems highly unlikely that knowledge of treatment might influence the levels of the measured biomarkers. Dr. Antonic confirmed there was no blinding (mail 2016-12-01).
Blinding of outcome assessment (detection bias)	Low risk	Dr. Antonic confirmed there was no blinding (mail 2016-12-01). However, since our outcome is a laboratory measure, we judged that the possibility of performance and detection bias was low.
Incomplete outcome data (attrition bias)	Low risk	No description of drop-outs. The short trial duration and the fact that the 'hospital length of stay' was measured for all included patients suggest no drop-outs. No drop-outs comfirmed by Dr. Antonic (mail 2016-12-01)
Selective reporting (reporting bias)	Low risk	The study protocol is not available. However, Tn is currently the most essential assay for cardiac damage and it does not seem reasonable to assume that Tn is selected from a large set of cardiac biomarkers, in particular as the result is negative.

Basili 2010

**Methods**	Randomized, double-blind, placebo-controlled trial, Sep 2007 to Dec 2008.<br>**3 papers describe different outcomes from this trial:**<br>Basili 2010:<br>https://pubmed.ncbi.nlm.nih.gov/20170881/<br>DOI: https://doi.org/10.1016/j.jcin.2009.10.025<br>Basili 2011:<br>https://pubmed.ncbi.nlm.nih.gov/21636808/<br>DOI: https://doi.org/10.1161/ATVBAHA.111.227959<br>Pignatelli 2011:<br>https://pubmed.ncbi.nlm.nih.gov/20629665/<br>DOI: https://doi.org/10.1111/j.1755-5922.2010.00168.x
**Participants**	Italy, elective PCI patients, 47 M / 9 F; age: vit C 66 (SD 8) y / control 68 (SD 9) y; 28 vit C / 28 control<br>**Inclusion**<br>>18 yr with clinically stable class I or II effort angina pectoris, a positive functional study for myocardial ischemia and a single *de novo* lesion in a native coronary artery that was scheduled for elective PCI.<br>**Exclusion**<br>Contraindication to aspirin or clopidrogel, previous MI, graft vessel disease, low platelet count, history of bleeding diathesis, renal dysfunction (from Pignatelli).
**Interventions**	**Vitamin C:**<br>Intravenous 1 g vitamin C over 1 h before PCI.<br>**Placebo:**<br>Saline.

**Outcomes**	TnI<br>8-OHdG plasma levels, at 1 h and 2 h<br>8-iso-PGF2a plasma levels, at 1 h and 2 h
**Timing of cardiac enzyme measurement**	Baseline and every 6 h over 2 days after PCI, and thereafter if these were abnormal values.
**Notes**	Only the median absolute increase in TnI from baseline was reported. We were able to contact Prof Violi for the baseline and 6 h troponin data, but data was not available any more (email 2020-11-3). We assumed that the baseline TnI levels in the vitamin C and control patients were balanced, and therefore the increase from baseline is a valid measure of the difference between the two groups.<br><br>**TnI:**<br>Troponin means were calculated from the reported medians and IQR in Basili 2010 p225 and Pignatelli 2011 p389:<br>VitC: median 0.008 [IQR 0.002-0.013];<br>Control: median 0.027 [IQR 0.005-0.032]<br><br>These give means: VitC: 0.0077; Control: 0.0213<br><br>The reported lower quartile level was 0.02 and 0.05 but that has to be an error since lower IQR limit cannot be greater than median. We assumed a typographical error so that one zero had been dropped.<br>We used the reported p-value of 0.0832 to calculate Z = 1.73, from which we estimated the SE(TE).<br><br>See the Excel file for the calculations and page 3 of this document.

Risk of bias table

Bias	Authors' judgement	Support for judgement
Random sequence generation (selection bias)	Low risk	"All eligible patients were randomly assigned in a 1:1 manner" (Basili 2010, p223).<br>"a computer-generated random sequence were used for randomization" (Pignatelli 2011, p387).<br>Balanced baseline demographic and clinical characteristics between groups (Basili 2010: Table 1).
Allocation concealment (selection bias)	Low risk	"opaque envelopes containing a computer-generated random sequence were used for randomization" (Pignatelli 2011, p387).<br>See also below.
Blinding of participants and personnel (performance bias)	Low risk	"The operators who performed the (angiographic) evaluation were unaware of the study protocol and of the patient's characteristics" (Basili 2010, p224).<br>"Laboratory analysis: To ensure blind analysis, cardiologists sent tube identified by numerical code to the laboratory where biologists perform analytical tests. The randomization list was unveiled after that the analytical phase was terminated"

		(Pignatelli 2011, p387; also mentioned in Basili 2011, p1767).
Blinding of outcome assessment (detection bias)	Low risk	See above.
Incomplete outcome data (attrition bias)	Low risk	"the enrolled patients completed all phases of the study" (Basili 2010, p224), which indicates no drop-outs. In addition, 1:1 randomization and reporting 28+28 patients is consistent with no drop-outs. This was confirmed by Prof. Violi (2020-03-13).
Selective reporting (reporting bias)	Low risk	"Serum cardiac-Troponin I (cTpI) was measured at baseline before the procedure, every 6 h over the next 2 days, and thereafter if these were abnormal values" (Basili 2010, p223). However, Basili does not describe what were the time points for which they reported the troponin values. The TnI values were published even though the result was negative.

Demirag 2001

**Methods**	Controlled trial<br>https://pubmed.ncbi.nlm.nih.gov/11281049/<br>https://www.mv.helsinki.fi/home/hemila/CAF/Demirag2001.pdf<br>DOI: https://doi.org/10.5281/zenodo.8287477
**Participants**	Turkey, CABG patients, 24 M / 6 F, age: 63.8 (SD 8.5) y, 10 vit C / 10 control, a third group (diltiazem) also had 10 patients, but is not included.<br>**Inclusion:**<br>Patients undergoing elective CABG.<br>**Exclusion:**<br>Recent MI, angina resistant to medical treatment,Ca channel blockers, medication with antioxidant properties, LVEF < 40%, additional organ dysfunction.
**Interventions**	**Vit C:**<br>0.05 g/kg of intravenous vitamin C just after the induction of anaesthesia and just before aortic declamping. Thus, the total dose was 0.1 g/kg. Assuming a 65 kg patient, this corresponds to 6.5 g/day on a single day.<br>**Control**:<br>Not described (Group C).
**Outcomes**	CKMB<br>Malonyldialdehyde (MDA) levels, measured before induction, after coronary sinus catheterization, after declamping, and after protamine administration.
**Timing of cardiac enzyme measurement**	Baseline and 2h after surgery
**Notes**	Table 2:<br>**CKMB:**<br>VitC: 68.6 SD 16.3; Control: 74.1 SD 21.0<br><br>There was also a third group of patients who received vitamin C together with diltiazem. This group was excluded from our analysis.

Risk of bias table

Bias	Authors' judgement	Support for judgement
Random sequence generation (selection bias)	Low risk	'The patients were randomly divided into three groups, 10 patients each'. (p69). The report published 10 + 10 + 10 which indicates that all randomized participants were included. The baseline age, LVEF, grafts/patient were balanced (Table 1).
Allocation concealment (selection bias)	Unclear risk	Not described
Blinding of participants and personnel (performance bias)	Low risk	Not described. It seems highly unlikely that knowledge of treatment might influence the levels of the measured cardiac enzymes.
Blinding of outcome assessment (detection bias)	Low risk	See above.
Incomplete outcome data (attrition bias)	Unclear risk	No flowchart, no description of any dropouts.
Selective reporting (reporting bias)	Low risk	The study protocol is not available The goal of the authors was “to compare the protective effects ...on ischaemia-reperfusion injury” (p68). CKMB was a standard test for evaluating possible cardiac damage at that time and was published. It seems implausible that CKMB was selected from a large set of cardiac markers.

Dingchao 1994

**Methods**	Parallel group trial.<br>https://pubmed.ncbi.nlm.nih.gov/7863489/<br>https://doi.org/10.1055/s-2007-1016504
**Participants**	China, CPB patients, adults, but age and sex not reported, 45 vit C / 40 control.<br>**Inclusion**<br>Patients undergoing cardiopulmonary bypass (CPB)<br>**Exclusion**<br>Not described.
**Interventions**	**Vit C**<br>Intravenously 0.125 g/kg 30 min before CPB and 0.125 g/kg at aortic declamping, indicating a total dose 0.25 g/kg. Assuming a 65 kg patient, this corresponds to 16.25 g/day on a single day.<br>**Control**<br>Not described, suggesting no treatment for the control group.
**Outcomes**	CKMB<br>Malonyldialdehyde (MDA) levels, measured at the same time points as CKMB (see below).
**Timing of cardiac enzyme measurement**	Before, during and several time points after the operation (13 times in total).

**Notes**	We measured the peak CKMB levels and their dispersion from Figure 1 by using the GIMP graphics program. However, we concluded that the dispersion parameter in the curves is SE, not SD as mentioned in the paper (last sentence method section). We recalculated the p-value of the difference in CKMB levels at 90 minutes (non-significant in the paper) while assuming that the reported SD is SE (scenario 1), and while assuming that SD is SD (scenario 2), see Additional File 2. The p-value of scenario 2 was very small, and the p-value of scenario 1 was close to what is expected from the curve. This was also concluded for the p-value of the difference at 120 min when assuming SD is SE.<br><br>Therefore, the peak CKMB levels and SE were measured from Figure 1. SE was then used to calculate the SD. See Additional File 2.<br><br>**CKMB:**<br>VitC (2h post-op): 38.68 SD 70.88; Control (4h post-op): 86.44 SD 101.6<br><br>We were unable to contact Dr. Dingchao through Research Gate (2021-5-6) and by mail (2022-1-7). Type of cardiac surgery is unknown but CPB is accompanied by ischemia/reperfusion phenomenon.

Risk of bias table

Bias	Authors' judgement	Support for judgement
Random sequence generation (selection bias)	Low risk	”There were no significant differences in age, weight, CPB time, and aortic clamping time between the two groups” (p276) The variables are not shown, however. Baseline malondialdehyde, CK, CKMB, LDH were all closely similar at baseline (Fig. 1)
Allocation concealment (selection bias)	Unclear risk	See above.
Blinding of participants and personnel (performance bias)	Low risk	Not described. However, it seems highly unlikely that knowledge of treatment might influence the levels of the measured cardiac enzymes.
Blinding of outcome assessment (detection bias)	Low risk	See above.
Incomplete outcome data (attrition bias)	Unclear risk	No flowchart, no description of possible dropouts.
Selective reporting (reporting bias)	Low risk	The study protocol is not available. However, LDH and CKMB were standard tests for evaluating possible cardiac damage at that time and both were published. Therefore, it seems implausible that CKMB was selected from a large set of cardiac markers.

Emadi 2019

**Methods**	Double-blind, placebo-controlled, randomized trial<br>https://pubmed.ncbi.nlm.nih.gov/31719005<br>http://www.ncbi.nlm.nih.gov/pmc/articles/pmc6852463
**Participants**	Iran, CABG patients, 32 M / 18 F, age: vit C 60 (SD 6) y, control 64 (SD 8) y, 25 vit C / 25 control.<br>**Inclusion:**<br>Patients who were referred to Shiraz hospitals for CABG.<br>**Exclusion:**<br>History of arrhythmias, LVEF < 30%, severe renal or hepatic failure, pacemaker, antiarrhythmic drugs or digoxin, AV-block or bradycardia < 50/min, recent MI, high initial TnI, and history of redo or complex cardiac surgery.
**Interventions**	**Vit C**<br>Intravenously 5 g IV vitamin C before anesthesia induction and 5 g of vitamin C in cardioplegic solution. Total dose 10 g/day on a single day.<br>**Control**<br>Saline as placebo.
**Outcomes**	TnI<br>CKMB
**Timing of cardiac enzyme measurement**	During induction of anesthesia, at the end of CABG, 6 h after surgery and 24 h after surgery.
**Notes**	Reported in Table 3:<br>**CKMB:**<br>VitC (24h post-op): 40.36 SD 22.79; Control (24h post-op): 53.80 SD 47.80<br>**TnI**<br>VitC (24h post-op): 0.811 SD 0.738; Control (24h post-op): 1.17 SD 1.92<br><br>We tried to contact Dr. Allahyari for the details about their methods but did not get response (2020-1-15, 2020-09-29 and 2021-3-16).

Risk of bias table

Bias	Authors' judgement	Support for judgement
Random sequence generation (selection bias)	Low risk	“Sample selection was done using a block randomization method” (p518). Age, sex, height, weight, LVEF, BP, Hb were balanced (Table 1). Number of grafts was also balanced (Table 2).
Allocation concealment (selection bias)	Low risk	'double-blinded' (p518). See also above.
Blinding of participants and personnel (performance bias)	Low risk	'double-blinded' (p518), but no details. Furthermore, it seems highly unlikely that knowledge of treatment by the patient might influence the levels of the measured biomarkers.
Blinding of outcome assessment (detection bias)	Low risk	'double-blinded' (p518), but no details.

Incomplete outcome data (attrition bias)	Low risk	No description of dropouts. The usage of block randomization, and the equal size of the 2 groups suggest no dropouts.
Selective reporting (reporting bias)	Low risk	The study protocol is not available. However, CKMB and Tn are current standard tests for evaluating possible cardiac damage and both were published. It seems implausible that they were selected from a large set of cardiac markers.

Oktar 2001

**Methods**	Controlled trial.<br>https://pubmed.ncbi.nlm.nih.gov/11768322/<br>https://doi.org/10.1080/003655101753267982
**Participants**	Turkey, CABG patients, 20 M / 4 F, age: vit C 55 (SD 9) y / control 56 (SD 9) y, 12 vit C / 12 control<br>**Inclusion:**<br>CABG patients<br>**Exclusion:**<br>Not described.
**Interventions**	**Vit C:**<br>Intravenously 4 g of vitamin C just before the induction of anaesthesia (Group II)<br>**Control**:<br>Not described, suggesting no saline as a placebo (Group IV).<br>(in Group III 4 g of vitamin C was added to the cardioplegic solution)
**Outcomes**	CKMB<br>Malonyldialdehyde (MDA) levels, measured Preoperative, Induction, PreCPB, After declamping, Post CPB, Skin closure, Postoperative and 1st day
**Timing of cardiac enzyme measurement**	Preoperative, induction, preCPB, after declamping, post CPB, skin closure, postoperative 0 h, 6 h, 12 h, 24 h, 36 h, 48 h, 72 h
**Notes**	In our study, we compared group II with group IV. There is another vitamin C group (group III) who were administered 4 g of vitamin C in the cardioplegic solution. We used group II because vitamin C was administered earlier compared to group III, and earlier administration can lead to more effective body distribution.<br><br>In our manuscript we also compared groups II and III against the control group IV. Furthermore, in a sensitivity analysis we carried out a meta-analysis of the effect of vitamin C on CKMB using the vitamin C group III. Results did not differ considerably, see printouts of statistical calculations at page 5 of this document.<br><br>Oktar (2001) study also included a fourth group I, which was administered vitamin E, which is not included in our analysis.<br><br>CKMB levels were extracted from Table 2. We concluded that the dispersion of CKMB Table 2 was SE and not SD, though SD was reported. In Additional File 2 we recalculated the p-values of the differences in CKMB at 48h (group II-IV) while assuming SD is SE (scenario 1) and SD is SD (scenario 2). The p-value of scenario 2 was very small (1,0E-09), whereas the p-value of scenario 1 (p=0.08) was close to the reported p-value of 0.03. This was also concluded for the

	p-value of the difference at 36h (group II-IV) when assuming SD is SE. Therefore, the reported dispersion of the CKMB results were considered to be SE's. The SE was used to calculate the SD. See Additional File 2.<br><br>**CKMB levels from the peaks of the curves:**<br>VitC group II (12h): 64.00 SD 32.5; Control (6h): 89.33 SD 26.2<br><br>We tried to contact Dr. Oktar for details of their methods but did not get any response (2021-3-16).

Risk of bias table

Bias	Authors' judgement	Support for judgement
Random sequence generation (selection bias)	Low risk	Groups were balanced for age, sex, NYHA, baseline LVEF, number of grafts, total bypass time (table 1). Baseline CKMB and MDA were all closely similar (Table 2).
Allocation concealment (selection bias)	Unclear risk	Not described. See above.
Blinding of participants and personnel (performance bias)	Low risk	Not described. Nevertheless, it seems highly unlikely that knowledge of treatment might influence the level of CKMB.
Blinding of outcome assessment (detection bias)	Low risk	See above.
Incomplete outcome data (attrition bias)	Low risk	No description of dropouts. The equal size of the 4 groups suggests that there may have been equal allocation to the 4 groups without dropouts.
Selective reporting (reporting bias)	Low risk	The study protocol is not available. CKMB was standard test for evaluating possible cardiac damage at that time. Given that the “study was undertaken to evaluate the effects … on markers of myocardial reperfusion injury and myocardial contractile function in coronary artery surgery” (p622), it seems implausible that CKMB was selected from a large set of cardiac markers.

Wang 2014

**Methods**	Randomized, placebo-controlled trial, Jun-Dec 2010.<br>https://pubmed.ncbi.nlm.nih.gov/24365194<br>https://doi.org/10.1016/j.cjca.2013.08.018
**Participants**	China, PCI patients; 374 M / 158 F; mean age: vit C 58 (SD 10) y / control 58 (SD 10) y; 265 vit C / 267 placebo<br>**Inclusion:**<br>Elective PCI for de novo lesions in native coronary arteries.<br>**Exclusion:**<br>Acute ST-segment or non−ST-segment elevation MI with an elevated baseline CKMB or Tn, cardiogenic shock, severe congenital or valvular heart disease that required surgical treatment, previous coronary stent implantation or CABG, and CKD.

**Interventions**	**Vitamin C treatment group:**<br>Intravenously 3 g vitamin C administered 2 to 6 h before procedures<br>**Placebo:**<br>Saline.
**Outcomes**	TnI<br>CKMB<br>8-OHdG plasma levels (ng/mL). Measured at baseline and 6 h after PCI.
**Timing of cardiac enzyme measurement**	Baseline, 6 h and 24 h after PCI
**Notes**	**CKMB:**<br>CKMB means were calculated from the reported medians and IQR in Table 1:<br>VitC: median 4.9 [IQR 4.1-5.7]; Control: median 6.1 [IQR 4.4-6.4]<br>These give means: VitC: 4.9; Control: 5.633<br><br>We used the reported p-value of 0.001 to calculate Z = 3.29, from which we estimated the SE(TE).<br><br>**TnI:**<br>Troponin means were calculated from the reported medians and IQR in Table 1:<br>VitC: median 0.03 [IQR 0.03-0.06]; Control: median 0.04 [IQR 0.02-1.12]<br>These give means: VitC: 0.04; Control: 0.393<br><br>We used the reported p-value of 0.017 to calculate Z = 2.39, from which we estimated the SE(TE).<br><br>See the Excel file for the calculations and page 3 of this document.<br><br>The Wang trial has by far the greatest weight in the CKMB analysis since it has 532 participants. In contrast, the total number of participants in the four other trials in Figure 3 is only 195. Therefore, we carried out a sensitivity analysis in which we excluded the Wang et al trial. The pooled effect of vitamin C on CKMB level remained significant: p=0.008, see printouts of statistical calculations at page 5 of this document.<br><br>We tried to contact Dr. Wang to ask details about their methods of blinding, but did not receive any response (2020-7-7, 2021-5-6).

Risk of bias table

**Bias**	**Authors' judgement**	**Support for judgement**
Random sequence generation (selection bias)	Low risk	"randomization was performed by a 1:1 ratio using computer-generated random numbers." (p97)<br>Groups were balanced for all clinical and angiographic characteristics (Supplemental Table 1 and 2).
Allocation concealment (selection bias)	Low risk	"All the operators were blinded to the randomization of the study." (p97)

Blinding of participants and personnel (performance bias)	Low risk	"All the operators were blinded to the randomization of the study." (p97)<br>Blinding of the participants wasn't described. However this would not have influenced the outcomes: it seems highly unlikely that knowledge of treatment by the patient might influence the level of TnI or CKMB.
Blinding of outcome assessment (detection bias)	Low risk	"All the operators were blinded to the randomization of the study." (p97)
Incomplete outcome data (attrition bias)	Low risk	Figure 1 shows that 614 patients were randomized to the vitC (N = 307) and control (N = 307) groups. After randomization 42 patients from the vitC group and 40 patients from the control groups were excluded, because they received drug treatment or CABG, and not PCI. However, the number of exclusions is balanced and there is no basis to assume bias caused by the exclusions.
Selective reporting (reporting bias)	Low risk	The study protocol is not available. However, the goal of the trial was "to assess whether preprocedural infusion of vitamin C is effective for prevention of PMI [periprocedural myocardial injury]" (p96), and CKMB and Tn are current standard tests for evaluating possible cardiac damage and both were published. It seems implausible that they were selected from a large set of cardiac markers.

Footnotes

Characteristics of excluded studies

Gültekin 2021

**Reason for exclusion**	The Gültekin (2021) paper has a substantial number of identical data with the paper of Demirag (2001). See: https://pubpeer.com/publications/8150B66EC0289FA4BF7C9ADB1C48A9#1. Therefore this trial cannot be included.

Moludi 2020

**Reason for exclusion**	The Moludi (2020) paper raises multiple serious concerns. See: https://pubpeer.com/publications/2E9E504061A7DC2AF6B2692D8632F1#1. Therefore this trial cannot be included.

Rostami 2016

**Reason for exclusion**	The Rostami (2016) paper described a potentially relevant outcome for our study in Table 5: 'Frequency distribution of positive serum troponin level on the first, second, and third day after'. However we don't know what 'positive' troponin means. This has not been described in the superficial method section. Therefore, the reported data cannot be used in our study. We contacted three authors (Dr. Rostami, Dr. Sharifi and Dr. Kamali) on 2021-08-31, 2021-09-07 and 2021-09-08 but unfortunately no response of Dr. Rostami.

Shafaei-Bajestani 2019

**Reason for exclusion**	The Shafaei-Bajestani (2019) paper raises multiple serious concerns. See: https://pubpeer.com/publications/1B59AD863839D55215841C1124C2D3#1. Therefore this trial cannot be included.

Footnotes
